# Advanced Detection of Failed LEDs in a Short Circuit for Automotive Lighting Applications

**DOI:** 10.3390/s24092802

**Published:** 2024-04-27

**Authors:** Jose R. Martínez-Pérez, Miguel A. Carvajal, Juan J. Santaella, Nuria López-Ruiz, Pablo Escobedo, Antonio Martínez-Olmos

**Affiliations:** 1 R&D Department, Valeo, 23600 Martos, Spain; jose-ramon.martinez-perez@valeo.com (J.R.M.-P.); juan-jose.santaella@valeo.com (J.J.S.); 2 Department of Electronics and Computer Technology, Escuela Técnica Superior de Ingenierías Informática y de Telecomunicación (ETSIIT), University of Granada, 18014 Granada, Spain; carvajal@ugr.es (M.A.C.); nurilr@ugr.es (N.L.-R.); pabloescobedo@ugr.es (P.E.)

**Keywords:** automotive, lighting, short-circuit detection, LED, diagnostic, neural network

## Abstract

This paper addresses the issue of LED short-circuit fault detection in signaling and lighting systems in the automotive industry. The conventional diagnostic method commonly implemented in newer vehicles relies on measuring the voltage drop across different LED branches and comparing it with threshold values indicating faults caused by open circuits or LED short circuits. With this algorithm, detecting cases of a few LEDs short-circuited within a branch, particularly a single malfunctioning LED, is particularly challenging. In this work, two easily implementable algorithms are proposed to address this issue within the vehicle’s control unit. One is based on a mathematical prediction model, while the other utilizes a neural network. The results obtained offer a 100% LED short-circuit fault detection rate in the majority of analyzed cases, representing a significant improvement over the conventional method, even in scenarios involving a single malfunctioning LED within a branch. Additionally, the neural network-based model can accurately predict the number of failed LEDs.

## 1. Introduction

Since the last years of the 20th century, the usage of solid-state lighting (SSL) technology in automotive lighting systems has led to a dramatic increase in performances and aesthetics in the final product [1]. Nevertheless, this also entails an exponential increase in design complexity compared to previous halogen-based systems [2]. Examples of hardware (HW) and software (SW) blocks utilized in automotive lighting systems include switch-mode power supplies (SMPSs) like DC/DC converters, high-performance light-emitting diodes (LEDs), communication buses, advanced control systems, redundant safety systems, and complex electronic control drivers [3,4]. Despite these advancements, regulations for automotive norms remain valid and apply similarly to simple halogen systems [5,6]. Therefore, there is a need to develop new, advanced techniques to cope with diagnosis rules while SSL devices and complex electronic modules are incorporated into design state-of-the-art (SoA) automotive products [7].

A typical topology of an automotive lighting application, whether mid-power (10–20 W or 150–500 mA) or high power (20–50 W or 500–1500 mA), consists of several connected LEDs in series, biased by a current-regulated DC/DC driver [8,9,10,11,12]. The DC/DC driver adapts the input voltage received from the vehicle (typically 12 or 24 V) to the current required to bias the LED branch. The output voltage can be lower, equal to, or higher than the input voltage, so a step-down/step-up converter is required. Typically, the maximum number of LEDs is up to 15 units. Typical currents used in lighting applications, such as low beam or high beam, or in signaling applications, like daytime running lights or turn indicators, range from 100 to 1500 mA. Sensors such as thermistors, to acquire the application’s actual temperature and the flux bin resistor (R_bin_), used to manage different efficiency LED classifications [13,14,15], are included in the lighting application.

Diagnosis is a feature that enables the electronic control unit (ECU) to communicate the function’s status to the vehicle, especially if there is any malfunction, sending the actual status to the cockpit, allowing the end user to make decisions on how to proceed. Moreover, for specific functions, particularly safety-critical ones [16], like turn indicators, diagnosis is mandatory by law [17,18].

These LEDs typically have a forward voltage (*V_f_*) ranging from 2 to 4 V at room temperature and a nominal current. However, this nominal voltage can be affected by several factors during the operation of the system, including voltage dispersion in nominal conditions due to LED parameter constructions, temperature, current, and aging.

Therefore, the expected forward voltage needs to be evaluated depending on the dynamic conditions. Additionally, the temperature for a given application may not be constant: thermal derating can be implemented to protect electronic components when running at high temperatures, or a low temperature to adapt the flux over temperature [19,20].

The current state-of-the-art method for LED diagnosis in automotive lighting systems is based on static calculations of upper and lower diagnosis threshold voltage values [21,22]. As long as the application returns an operating voltage between these thresholds, the system will be considered as OK. However, typically, the gap is too large to detect a single short-circuited LED, thus several failed LEDs may remain undetected.

In this work, several dynamic algorithms are proposed to consider the actual operating conditions of a lighting system and improve the accuracy of the diagnosis method in order to detect a single LED in a short circuit. The novelties and contributions introduced by the presented work are as follows:

1. Two different algorithms are proposed, one based on a mathematical model and another on a neural network, for detecting a single LED malfunction due to a short circuit within a lighting branch.
2. Both algorithms exhibit an accuracy rate above 99% in all studied cases of a single LED short circuit, contrasting with the technique currently used for lighting branch diagnosis, which is incapable of detecting it.
3. The neural network is capable of detecting not only the presence of LEDs in a short circuit within the branch, but also determining the number of faulty elements.
4. The neural network can be trained with either real experimental data or data obtained through simulations using information provided by LED manufacturers, providing a large dataset for training without the need for experimental tests.
5. Both developed methods are optimized to obtain simple algorithms that result in a reduced computational overhead for the microcontroller controlling the lighting system’s operation.
6. Neither of the developed algorithms require new elements in the lighting board design; they utilize already available resources.

## 2. Technical Background

### 2.1. State of the Art: LED Failure Detection via Threshold

As outlined in Section 1, the usual method for detecting LED short circuits in lighting and signaling applications is through a threshold voltage. During operation, the system measures the direct voltage across the LED branch; as long as the measured voltage is between the upper and lower thresholds, the system is deemed correct. If the measured voltage falls below the lower voltage threshold, the system reports a short-circuit error and is designated as “not OK” (nOK) [21]. If the measured voltage is above the upper voltage threshold, an open-circuit failure is predicted. Open-circuit errors are easily identifiable as the bias current is zero in such instances. The upper and lower voltage thresholds are statically calculated for a specific application, considering absolute calculations for both the worst- and best-case scenarios.

The upper and lower voltage limits that define the proper operation of an LED branch (*V_fnLim_*) are calculated according to Equation (1):

(1) $$V_{fn_{Lim}}=n\cdot \left(V_{f_{Lim}}+\Delta V_{f}(T_{Lim})+\Delta V_{f}(I_{Lim})\right),$$

where *n* is the number of LEDs in the branch, *V_fLim_* is the upper or lower limit value of the nominal voltage drop for the considered LED model, ∆*V_f_*(*T_Lim_*) is the variation in this nominal voltage due to thermal drift evaluated at the upper or lower temperature limits (−40 °C and 150 °C for automotive electronics), and ∆*V_f_*(*I_Lim_*) is the variation in the LED nominal voltage due to changes in the bias current.

These voltage limits are statically calculated based on manufacturer-specified data for the LED model used in each branch for all units produced in the factory, without any dynamic adaptation once the system is operational.

### 2.2. Proposed Models for Failure Detection

As discussed in the previous section, fault detection in an LED branch in automotive applications is commonly performed through the voltage threshold or limit values method. This method accounts for variations in LED voltage values across the full range of temperature, bias current, and dispersion of production. The voltage range generated between the calculated lower and upper limit values, as per Equation (1), can conceal the failure of one or more LEDs within the branch. This is because a small number of LEDs in short circuit within the branch can shift the voltage drop value of the entire branch within the calculated limits, and this error would not be detected using this method [23]. Furthermore, the limits for applying this method are calculated only once during the design process, and the obtained values are applied uniformly to all manufactured lighting units. This occurs without regard to variations among them or adaptation to operating conditions.

To enhance dynamic fault detection in the operation of one or more LEDs in a lighting or signaling branch in automotive applications, we propose two implementable methods in the vehicle’s control unit. These models are based on the periodic measurement of the voltage drop, temperature, and bias current in each LED branch during the system’s runtime. This information, combined with manufacturer-specified data for the LEDs comprising the lighting or signaling branches, allows the generation of dynamic models that adjust the voltage limits within the acceptable voltage drop range for an LED branch. In this way, a more precise detection of the failure of one or more LEDs can be achieved.

#### 2.2.1. Mathematical Model

The voltage drop in a branch with *n* LEDs (*V_fn_*) polarized with a constant current or in PWM mode has three main contributions: the nominal voltage drop of each individual LED (*V_f_*), the variation in this forward voltage due to changes in the bias current (∆*V_f_*(*I_B_*)), and the variation in this forward voltage produced by changes in the operational temperature (∆*V_f_*(*T*)) [24], as expressed in Equation (2):

(2) $$V_{fn}=\sum_{i=1}^{n}\left(V_{f_{i}}+\Delta V_{f_{i}}(T)+\Delta V_{f_{i}}(I_{B})\right)$$

Is we assume that the *n* LEDs in the branch are identical, Equation (2) can be rewritten as:

(3) $$V_{fn}=V_{fn_{0}}+\Delta V_{f_{n}}(T)+\Delta V_{f_{n}}(I_{B})=V_{fn_{0}}+n\Delta V_{f_{i}}(T)+n\Delta V_{f_{i}}(I_{B})$$

where *V_fn_*_0_ is the voltage drop in the branch under controlled conditions of temperature and bias current.

The nominal voltage drop, *V_fn_*_0_, can be determined through a calibration process during the manufacturing of the lighting system or at the end of line (EOL), when an external control unit verifies the proper operation of the lighting or signaling branch. Temperature and bias current can be periodically measured through sensors and a microcontroller. With these three values, a prediction of the voltage drop in a properly functioning LED branch, i.e., where all LEDs in the branch are producing illumination (referred to as the OK condition), can be generated following Equation (3). This prediction is compared with the measured voltage drop in the same LED branch; if there is a difference between both values greater than a predetermined threshold, the branch is considered to have one or more LEDs in a malfunctioning condition (nOK).

Therefore, in this model, an LED branch is considered to be in a malfunctioning state (nOK) if the predicted voltage drop under proper operation (*V_fn_*_0_) exceeds the measured voltage drop (*V_fnmeas_*). This indicates that a short-circuit failure has occurred in at least one of the LEDs, as expressed in Equation (4):

(4) $$if\;\frac{V_{fn_{0}}-V_{fn_{meas}}}{V_{fmin}}>\delta \to \;nOK,$$

where *δ* is a threshold voltage value that is set close to the minimum nominal voltage (*V_fmin_*) of a single LED and is continuously updated with periodic measurements of the bias current and temperature. Its value is experimentally adjusted to compensate for limitations and tolerances in the hardware components and software code.

#### 2.2.2. Neural Network-Based Model

As an alternative method proposed in the previous section (mathematical model), we suggest the use of an Artificial Neural Network (ANN), a tool commonly used for classification and error diagnosis in LED-based lighting systems [25,26].

ANNs are mathematical models inspired by biological neural networks, in which each processing unit (neuron), with *m* inputs, calculates its output, *a*, as expressed in Equation (5) [27]:

(5) $$a=f\left(\sum_{i=1}^{m}w_{i}\cdot p_{i}+b\right),$$

where *w_i_* and *p_i_* are the inputs to the current neuron and *b* is the bias, *f* being the activation function of the ANN. The weights and biases of a given neural network are calculated starting with a dataset to optimize the expected result (supervised learning).

In the proposed case in this study, the inputs to the ANN are: the number of LEDs in the branch (*n*), actual measured voltage, current, and temperature (*V_fn_*, *I_B_*, *T*), as well as the measured voltage, current, and temperatures at the time of calibration (*V_fn_*_0_, *I_B_*_0_, *T*_0_).

To train the neural network, a dataset is constructed with inputs corresponding to situations of the LED branch, whose state (OK or nOK) is known, obtained either through simulations or real measurements. These inputs, along with their corresponding states, are introduced to the ANN for the process of supervised learning. 

After training the neural network, the values of *w*, *p*, and *b* for the different neurons in the chosen topology are integrated into the vehicle’s control unit to implement the ANN. This will periodically run, updating the measured inputs of voltage, current, and temperature, and will return the state (correct or malfunction) for each of the analyzed LED branches.

## 3. Materials and Methods

The LED failure detection algorithms described in Section 2 were tested to assess their performance in three different configurations.

- A signaling system with a branch composed of 12 white LEDs polarized with a nominal current of 150 mA.
- A signaling system with a branch composed of 6 white LEDs polarized with a nominal current of 150 mA.
- A lighting system with a branch composed of 7 white LEDs polarized with a nominal current of 750 mA.

These systems used the LED models Nichia NCSW170DT (Nichia Corporation, Tokushima, Japan) and Cree AHG (Cree, Inc., Durham, NC, USA).

**Figure 1:**
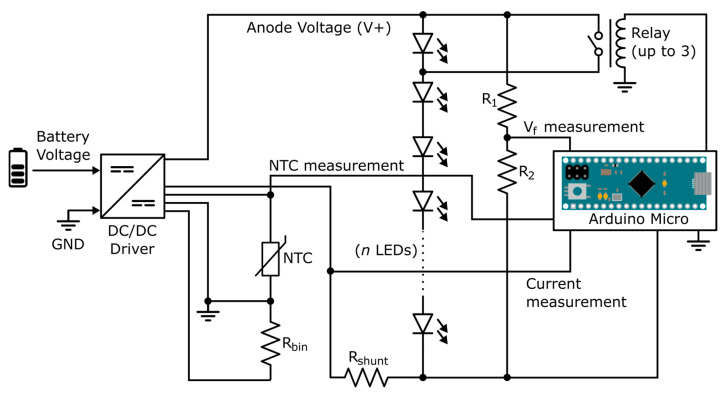
Scheme of the experimental setup.

The experimental setup for algorithm testing is schematized in Figure 1. A DC/DC driver was used to generate the bias current for the analyzed branch, consisting of 12, 6, or 7 LEDs. An Arduino Micro development platform (Arduino, Somerville, MA, USA) based on an ATmega32U4 microcontroller (Microchip Technology Inc., Chandler, AZ, USA), served as the control unit and periodically measured the voltage drop values in the LED branch, bias current, and temperature through NTC thermistor model NCP108X103xSRB (Murata Manufacturing Co., Ltd., Nagaokakyo, Tokyo, Japan). Additionally, a set of three relays controlled by the microcontroller was included to emulate the short-circuit failure of 1 to 3 LEDs in the branch.

Climatic chamber model CTS T 70/600 (CTS GmbH, Kall, Germany) was employed, capable of creating stable temperature conditions ranging from −70 °C to 180 °C, to vary the operating temperature of the tested system.

**Figure 2:**
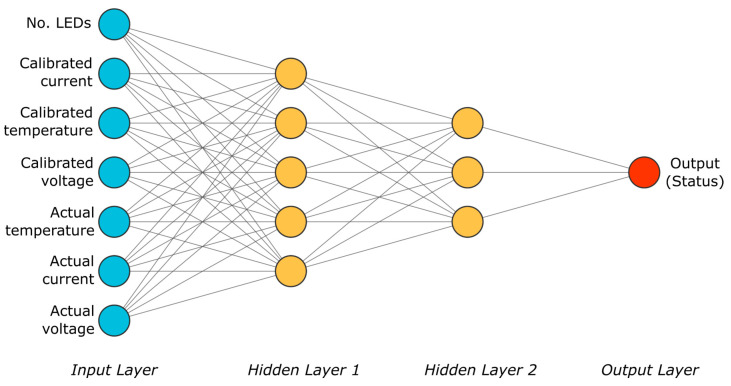
Neural network scheme.

A neural network with 4 layers, depicted in Figure 2, was designed. It comprises 7 inputs corresponding to the number of LEDs in the branch (*n*), calibration variables (*V_fn_*_0_, *I_B_*_0_, *T*_0_), and real-time variables during execution (*V_fn_*, *I_B_*, *T*), and 1 output ranging from −2 to 1. During neural network training, an output of −2 was assigned to a situation where there were 3 LEDs short-circuited in the branch, −1 for 2 LEDs short-circuited, 0 for 1 failure, and 1 for a branch operating with all its LEDs functioning correctly. This topology was optimized in terms of simplicity to identify the configuration with the highest accuracy rate in diagnosing the lighting branch while minimizing computational costs.

If the generated output was below a specified threshold, *γ*, whose value is settled by the system developer or programmer, the LED branch status was predicted as nOK (indicating at least one LED in a short circuit); conversely, if the neural network output exceeded, γ, the branch status was predicted as OK.

A dataset comprising 20,000 entries was generated for training the neural network using a custom simulation code based on C programming language. Although the data generated for this training could be based on real measurements, in this case, they were generated using theoretical values of the voltage, current, and temperature of the LEDs provided in the manufacturer’s specifications.

The code to implement the two proposed LED failure detection methods was introduced into the microcontroller of the Arduino Micro board. The microcontroller executed the code continuously, updating the measured values of bias current, voltage drop in the branch, and temperature. The output of both codes was monitored through serial communication with a PC.

**Figure 3:**
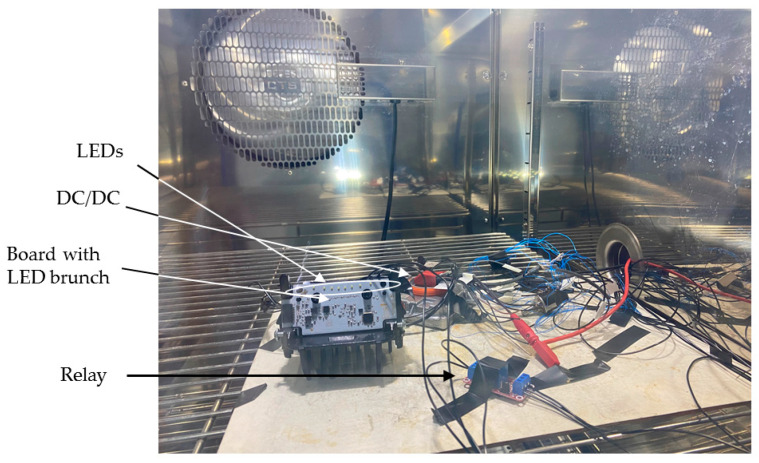
Experimental setup in the climatic chamber.

Calibration data were obtained at room temperature (25 °C ± 2 °C). The system underwent continuous evaluation within the climatic chamber, as shown in Figure 3, spanning from −40 °C to 105 °C over 8 h, with a slope of approximately 0.3 °C/min. This was conducted to observe the model’s behavior across the entire automotive temperature range. Three distinct LED boards were employed as replicas to assess potential component dispersion. Thermal protection was implemented by reducing power through a PWM signal. Additionally, a filtering algorithm was applied to obtain the necessary data, particularly the current and voltage.

## 4. Results

The fault detection methods for short circuits in signaling or lighting LED branches were tested in the system described in the previous section. For this purpose, three different types of branches with 6 (case A), 7 (case B), and 12 (case C) LEDs in series were used, polarized with a constant current of 150, 720, and 150 mA, respectively. For each branch model, three replicas were considered, using three different electronic boards.

In all experiments, approximately 3000 executions were performed with the temperature varying between −40 and 105 °C, as described in the previous section. In these executions, the relay sequentially changes to provoke the short circuit of one LED in the branch for cases A and B, thereby alternately emulating the branch in OK and nOK states with a single faulty LED. For case C, three relays were used to sequentially short-circuit 0 (no faults), 1 (single fault), 2, or 3 (multiple fault) LEDs in the branch.

### 4.1. Success Rate

After configuring the relays, measurements of current, branch voltage, and temperature were taken, and it was predicted whether the branch was functioning correctly or malfunctioning using both the classical threshold-based detection method and the algorithms proposed in Section 2.2. For case C, the neural network was programmed with two different models resulting from training the network with a dataset corresponding to one branch of LEDs with either zero or one failure (referred to as the single failure (SF) model), and another dataset corresponding to the same branch of LEDs with a number of errors between 0 and 3 (referred to as the multifailure (MF) model).

The results obtained are shown in Table 1, where the success rate in the prediction of both OK and nOK statuses in the analyzed scenarios is presented. Each proposed failure diagnosis algorithm (mathematical and NN-based) was tested using three different values of the thresholds *δ* and *γ* between 0 and 1. These values are proposed by the authors based on our knowledge and previous experience.

As depicted in Table 1, the classical method of short-circuit fault detection based on voltage thresholds is unable to detect the failure of a single LED in any of the analyzed cases. This outcome was expected since the voltage change in an LED branch caused by the short circuit of a single LED was small and did not cause the total voltage drop of the branch to exceed the thresholds established in Equation (1). The probability of detecting a malfunctioning LED branch increases with the number of LEDs in failure, as observed in the results obtained for case C. 

The error in detecting a single LED failure contributes to enhancing the robustness of the failure detection algorithm to comply with global regulations, ensuring a more consistent behavior, particularly in relation to ‘single lamp’ scenarios [28]. The diagnostic methods proposed in this work consistently exhibit a high success rate in both branches with a single LED short circuit and branches with multiple LEDs short-circuited, as reflected in the results presented in Table 1. For both the mathematical method and the neural network-based method, the best results are achieved when the threshold value (*δ* or *γ*) is set to 0.5, where the success rate is always above 99%, and in most of the studied cases, it reaches 100%.

The neural network-based diagnostic algorithm has been applied in a new test aimed not only at determining whether the LED branch operates correctly or contains any short-circuit faults, but also at predicting the number of malfunctioning LEDs. To achieve this, the output was discretized using three thresholds to establish the ranges shown in Table 2, corresponding to the situations of OK (0 faults), nOK-1 (1 fault), nOK-2 (2 faults), and nOK-3 (3 faults).

This configuration was applied to the data generated for case C in Table 1 (12-LED branch with a number of faults ranging from 0 to 3) using the neural network trained with multiple faults (MFs). The results in predicting the number of LEDs in short circuit are presented in Table 2.

Based on the findings presented in Table 2, it is evident that the neural network-based method proposed in this study demonstrates the ability not only to identify whether the LED branch contains short-circuited elements, but also to predict the number of malfunctioning LEDs with a reliability exceeding 99% in the studied examples.

### 4.2. Computational Effort

The diagnostic methods for LED branches must be executed on the microcontroller integrated into the driver, which has limited resources. Therefore, it is necessary to ensure that the computational load introduced by the short-circuit fault detection algorithms implemented is minimized and does not hinder the operation of this unit.

In Table 3, the number of CPU operations required for the implementation of each of the detection algorithms considered in this work is shown. In addition, the memory requirements for the storage of data and code associated to each diagnosis method are included.

To ensure compliance with the timing requirements specified in ISO 26262 safety regulations [17], the computational overhead introduced by the diagnostic algorithms must be sufficiently low to not significantly impact the system’s performance. Since ISO 26262 sets specific requirements for the functional safety of electronic systems in automotive applications, it is crucial that any additional computational overhead does not compromise the system’s ability to perform its critical safety functions within specified time constraints.

The execution time of these algorithms depends on the microcontroller integrated into the LEDs branch driver. The computational overhead introduced by these algorithms, which is calculated as the extra execution time that the microcontroller must work in each evaluation cycle, depends on the number of diagnostics performed per second.

For a mid-range microcontroller, such as an ATmega32U4 (16 MIPS @ 16 MHz), and assuming that the execution of these algorithms is carried out every 100 ms, the execution time and the computational overhead produced by each diagnostic method were obtained. The results are presented in Table 4.

As observed from Table 3 and Table 4, the classic diagnostic method based on voltage thresholds is the simplest to implement and produces the least overhead on the microcontroller, while the models proposed in this work reached overhead levels of 0.4% and 1.5% under the assumed conditions. This represents a significantly higher load than that produced by the classic method. However, this overhead does not result in a real detriment to the microcontroller’s efficiency. Additionally, the values obtained for the computational overhead can be reduced by increasing the execution cycle assumed to be 100 ms in this case. Furthermore, the use of a higher-performance microcontroller would also reduce the execution time of these diagnostic methods, as well as the computational overhead they entail.

## 5. Conclusions

In this work, two diagnostic methods for LED branches have been presented as alternatives to the algorithm typically implemented in the control units of vehicle lighting and signaling systems. The introduced algorithms, referred to as the mathematical model and the neural network-based model, have been tested on real systems with branches of 6, 7, and 12 LEDs, where short-circuit faults are emulated through relays, within a temperature range from −40 °C to 105 °C controlled in a climatic chamber. The obtained results have been compared with those corresponding to the traditional diagnostic method based on voltage thresholds.

The tests conducted have revealed that the conventional method of short-circuit fault detection based on voltage thresholds fails to reliably respond when the number of failed LEDs in the branch is low. Particularly notable is the case of a single LED short circuit, as this method is entirely incapable of detecting such a fault. The methods proposed in this work address this issue and achieve prediction accuracy rates for the branch state above 99%, even with a single malfunctioning LED within a broad branch of 12 LEDs. Furthermore, the neural network-based algorithm can predict, with a reliability exceeding 99%, how many LEDs in the branch will fail due to short circuits, which can be useful for estimating when a lighting or signaling unit should be repaired. It is worth noting that these results, in the case of the neural network-based method, have been obtained through training using data generated from the technical specifications of the LEDs, without including experimental measurements. This represents a significant advantage in training the network.

The proposed methods for LED fault detection are based on mathematical algorithms deliberately simplified to avoid excessive computational cost for the control units where they are implemented; the computational overload introduced by these diagnostic methods on the microcontroller integrated into the driver of each LED branch has been estimated. It has been concluded that, under typical execution cycles, this overload does not pose a critical burden on the microcontroller, being limited in the studied cases to 1.5% of the device’s capacity. This indicates that the developed methods are fully capable of being implemented in real automotive lighting and signaling units. Besides posing a reduced computational burden, they do not require new elements that would entail a change in the design of the control boards for LED branches. Instead, they make use of the devices already available in them. 

## Figures and Tables

**Table 1:** Failure detection success rates.

		Threshold Voltage Method	Mathematical Algorithm			NN-Based Algorithm		
Case Study	Branch Status		δ = 0.35	δ = 0.5	δ = 0.75	γ = 0.25	γ = 0.5	γ = 0.75
A (6 LEDs)	OK	100%	100%	100%	100%	100%	100%	100%
	nOK	0%	100%	100%	100%	93.67%	100%	100%
B (7 LEDs)	OK	100%	99.33%	100%	100%	100%	100%	78.67%
	nOK	0%	100%	100%	100%	99.23%	100%	100%
C (12 LEDs)	OK	100%	99.57%	100%	100%	100% SF 100% MF	100% SF 99.6% MF	89.2% SF 78.8% MF
	nOK: 1 F	0%	100%	100%	99.57%	99.46% SF 99.87 MF	100% SF 100% MF	100% SF 100% MF
	nOK: 2 F	81.67%	100%	100%	100%	100% SF 100% MF	100% SF 100% MF	100% SF 100% MF
	nOK: 3 F	100%	100%	100%	100%	100% SF 100% MF	100% SF 100% MF	100% SF 100% MF

**Table 2:** Number of failed LEDs’ prediction success rate.

Number of Failed LEDs	0	1	2	3
NN output range	[0.5, 1]	[−1, 0]	[−2, −1]	[−3, −2]
Success rate	99.6%	99.5%	99.8%	99.3%

**Table 3:** Number of operations required to implement the diagnosis algorithms.

Operation	Threshold Voltage Method	Mathematical Algorithm	NN-Based Algorithm
Integer sum	0	0	315
Integer multiplication	0	0	105
Floating-point sum	0	16	105
Floating-point multiplication	0	27	105
Floating-point comparison	1	1	1
EEPROM	62 bytes	1.1 kbytes	2.5 kbytes
Data storage	8 bytes	72 bytes	504 bytes

**Table 4:** Execution time and computational overhead.

	Threshold Voltage Method	Mathematical Algorithm	NN-Based Algorithm
Execution time (µs)	10	400	1500
Computational overhead (%)	0.01	0.4	1.5

## Data Availability

The datasets in this study may be made available upon reasonable request.
